# Routine Clinical Practice Treatment Outcomes of Eplerenone in Acute and Chronic Central Serous Chorioretinopathy

**DOI:** 10.3389/fphar.2021.675295

**Published:** 2021-05-10

**Authors:** Katrin Fasler, Jeanne M. Gunzinger, Daniel Barthelmes, Sandrine A. Zweifel

**Affiliations:** ^1^ Department of Ophthalmology, University Hospital Zurich, Zurich, Switzerland; ^2^ University of Zurich, Zurich, Switzerland; ^3^ Save Sight Institute, The University of Sydney, Sydney, NSW, Australia

**Keywords:** aldosterone antagonists, central serous chorioretinopathy, eplerenone, medical retina, retinal disease

## Abstract

**Purpose:** To evaluate efficacy of eplerenone therapy vs. observation on resolution of subretinal fluid (SRF) in patients with acute and chronic central serous chorioretinopathy (CSCR) in routine clinical practice.

**Methods:** Retrospective comparative case series of eyes diagnosed with CSCR treated with eplerenone or observation. Primary outcome measure was maximum height of SRF at 12 months. Secondary outcome was percentage of eyes with complete resolution of SRF, percentage of eyes with reduction of SRF ≥50%, and best corrected visual acuity (VA) at 12 months. Separate analysis was conducted for eyes with acute and chronic CSCR.

**Results:** Sixty-eight eyes of 60 patients (82% male) were included. Eleven of the 38 eyes with acute CSCR, and seven of the 30 eyes with chronic CSCR, received eplerenone. Subretinal fluid decreased from baseline to 12 months in acute (287 ± 221 to 31 ± 63 µm) and chronic (148 ± 134 to 40 ± 42 µm) CSCR. Kaplan-Meier curves were similar for treated and observed eyes and COX regression analysis did not show a significant difference in SRF resolution in treated vs. observed eyes (p = 0.6 for acute, p = 0.2 for chronic CSCR).

**Conclusion:** This routine clinical practice outcome study did not show evidence of efficacy of eplerenone on resolution of SRF in acute nor chronic CSCR.

## Introduction

Central serous chorioretinopathy (CSCR) is a retinal disease characterized by subretinal fluid (SRF) accumulation causing a neurosensory detachment and alterations of the retinal pigment epithelium (RPE) (Liew et al., 2013; Mehta et al., 2017). The precise pathophysiology of CSCR remains unknown, but advances in retinal imaging, particularly enhanced-depth optical coherence tomography (EDI-OCT) improved understanding of retinal and choroidal changes observed in CSCR. Increased choroidal thickness (pachychoroid) was correlated with CSCR, supporting the important role of choroidal abnormalities as underlying cause of RPE dysfunction and SRF leakage leading to neurosensory detachment (Dansingani et al., 2016). Recently, a new classification was proposed, embedding CSCR into the spectrum of pathology with increased choroidal vascularity index or “pachychoroid diseases” (Dansingani et al., 2016; Singh et al., 2019).

Two subtypes of CSCR are distinguished: Acute CSCR is usually self-limited and has a favorable visual prognosis (Liew et al., 2013). Chronic CSCR however, is often associated with progressive visual impairment due to persistent SRF and subsequent damage to the neuroretina and RPE resulting in atrophy of both (Loo et al., 2002).

There is no evidence-based consensus for the management of either acute or chronic CSCR. For acute CSCR, the most common initial approach is observation (Salehi et al., 2015; Mehta et al., 2017). For chronic CSCR, a variety of interventions has been proposed, among them aldosterone antagonists, subthreshold laser therapy, and photodynamic therapy (Salehi et al., 2015; Scholz et al., 2016; Cakir et al., 2019; Iacono et al., 2018; Zola et al., 2018; Fusi-Rubiano et al., 2019; Lee et al., 2019; Wang et al., 2019).

Preclinical studies reported that CSCR may result from over-activation of the mineralocorticoid receptor pathway in the choroid (Zhao et al., 2012; Daruich et al., 2015). Based on these findings, a pilot study demonstrated potential clinical efficacy of eplerenone (Bousquet et al., 2013). Following this, several studies assessed aldosterone-antagonist treatment with variable outcomes (Behar-Cohen and Zhao 2016; Bertan Cakir et al., 2016; Daruich et al., 2016; Ghadiali et al., 2016; Kapoor and Wagner 2016; Pichi et al., 2016; Rahimy et al., 2017; Schwartz et al., 2017). In 2019, the first sufficiently powered prospective study did not find a clinically significant benefit of eplerenone over placebo in chronic CSCR (Lotery et al., 2020b).

In this study, we evaluated eplerenone therapy vs. observation for acute and chronic CSCR in routine clinical practice.

## Materials and Methods

### Ethics

Institutional review board approval (Ethics Committee of the University of Zurich, BASEC-No. PB_2016-00264) was obtained and all patients gave informed consent to publish their clinical data. The study adheres to the tenets of the Declaration of Helsinki.

### Data Collection

Patients aged 18 years and older who were diagnosed with acute or chronic CSCR and were consecutively seen at the Department of Ophthalmology, University Hospital Zurich between July 2008 and March 2017, were included in this retrospective study. Diagnosis of CSCR was made by multimodal imaging: Spectral domain EDI-OCT, autofluorescence, fluorescein- and indocyanine green angiography. The distinction between acute and chronic CSCR was based on the duration of symptoms and signs of chronicity in multimodal imaging (symptoms ≥3 months and descending tracts/hypoautofluorescence on autofluorescence/outer retinal or pigment epithelium atrophy = chronic). Exemplary cases of acute and chronic CSCR are shown in Supplementary Figures S1,S2.

**FIGURE 1:**
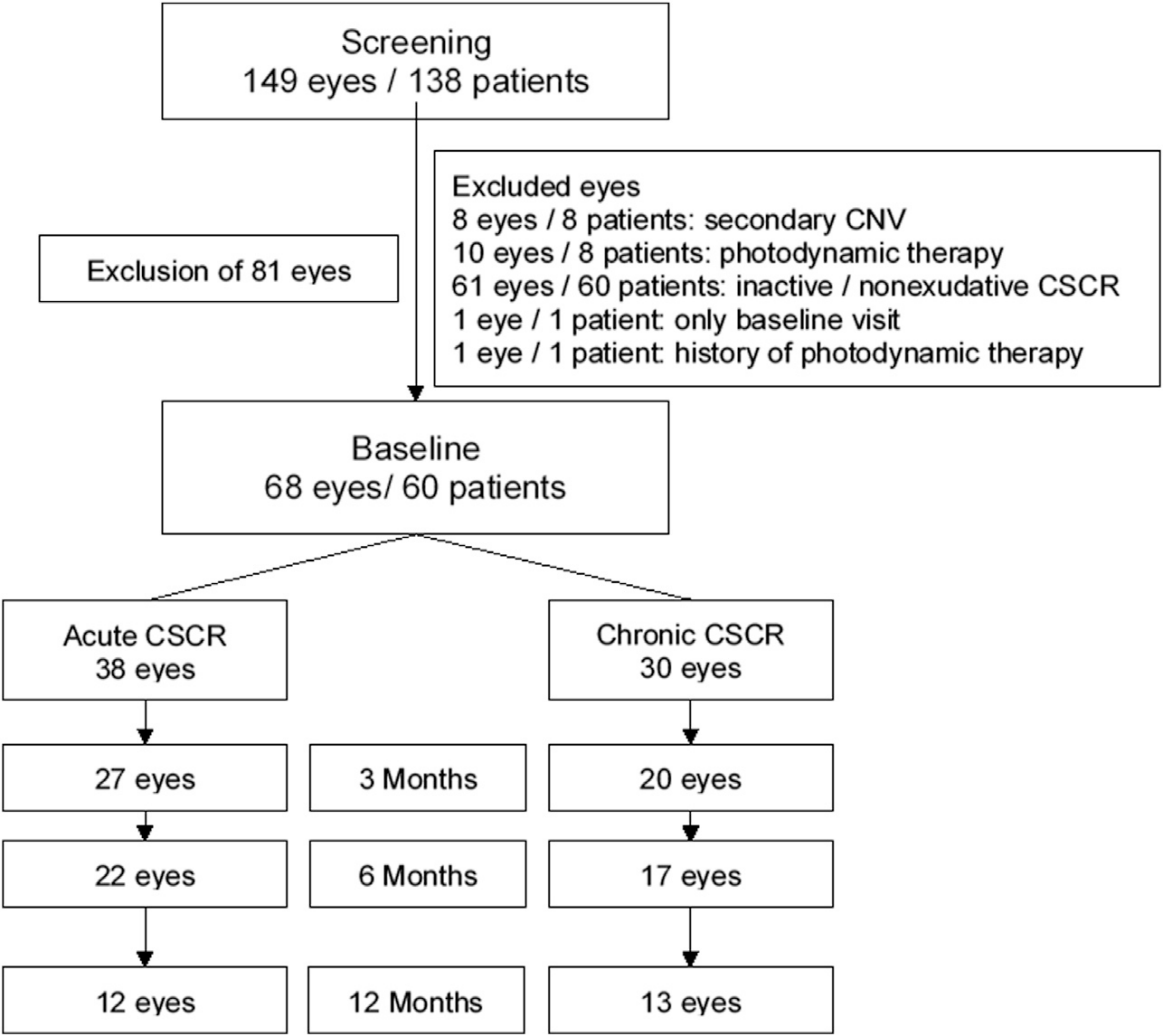
Consort style flow diagram of data collection and follow-up. CNV–choroidal neovascular membrane, CSCR central serous chorioretinopathy.

Exclusion criteria were secondary choroidal neovascularization, pachychoroid neovasculopathy, history of photodynamic therapy (PDT)/thermal laser/anti-VEGF therapy, or history of mineralocorticoid-antagonist therapy. Exogenous steroid therapy was not an exclusion criteria. Excluded were 8 eyes with secondary choroidal neovascularization (CNV), 10 eyes receiving PDT, and 61 eyes with signs of CSCR that never had any SRF during the observation period (inactive or so called non exudative CSCR), 1 eye with only baseline visit, and 1 eye with history of PDT treatment. Consort style flow diagram of data collection is shown in Figure 1. The duration of eplerenone therapy was measured in weeks. There was no matching of patients/eyes.

### Baseline and Outcome Measures

Baseline time point for the observation only group was defined as date of first presentation, for the treatment group as date of initiation of eplerenone therapy. There was a minimum follow-up of 48 weeks for SRF analysis at 12 months, but no minimum follow-up for survival analysis. Primary outcome measure was maximum height of SRF at 12 months as detected on EDI-OCT imaging. 12 month date was defined as date closest to 365 days after baseline of eyes having at least 48 weeks of follow-up. Secondary outcome was percentage of eyes with complete resolution of SRF and reduction of SRF ≥50% at 12 months, time to reach 0 SRF, and best corrected decimal VA at 12 months. Choroidal thickness could not be measured consistently due to errors in image centration at acquisition and indiscernible sclera-choroidal junction, precluding reasonable statistical analysis. Reasons for discontinuation of eplerenone were assessed.

### Imaging/Image Grading

Fluorescein angiography images were obtained using a Zeiss camera (FF450 Plus - Version 4.5.2) or Heidelberg Spectralis (Heidelberg Engineering, Heidelberg, Germany), EDI-OCT and autofluorescence images were obtained with Heidelberg Spectralis (version 1.9.13.0) and viewed with the contained Heidelberg software (Spectralis Viewing Module 6.5.2.0; Heidelberg Engineering, Heidelberg, Germany). Grading was carried out by a retina specialist (KF). SRF was measured in µm manually at the maximal level of the neurosensory detachment from the RPE to the outer border of the hyperreflectivity of the outer retinal layers perpendicular to the RPE.

### Statistics

Data were coded in Excel and analyzed in SPSS statistics software (IBM Corp. Released 2017. IBM SPSS Statistics for Windows, Version 25.0. Armonk, NY: IBM Corp). The eye was defined as unit of analysis. Mean values in text are expressed with ± standard deviation, median values with 95% confidence interval, unless otherwise specified. Kaplan Meier Curves and Cox Regression were used to assess the effect of eplerenone therapy on resolution of SRF.

## Results

### Demographics and Baseline Characteristics

Sixty-eight eyes of 60 patients (82% male) qualified for inclusion in this study. Details of in-/exclusion and number of eyes up to 12 months are shown in Figure 1. Thirty-eight eyes were considered to be acute and 30 eyes chronic CSCR. Mean age at presentation was 40 ± 10 (range 27–72) years for acute and 48 ± 8 (range 36–66) years for chronic CSCR patients. Mean follow-up time was 46 ± 45 weeks (range 1–188) for eyes with acute and 81 ± 90 (range 4–266) weeks for eyes with chronic CSCR. Eleven of the 38 eyes (29%) with acute CSCR, and 7 of the 30 eyes (23%) with chronic CSCR, received eplerenone 25 mg/day for 1 week followed by 50 mg/day. Mean therapy time was 24 ± 16 (range 7–50) weeks for patients with acute and 74 ± 90 (range 10–205) weeks for patients with chronic CSCR. Baseline characteristics are shown in table 1.

**TABLE 1:** Baseline characteristics and outcomes at 12 months of eyes with acute and chronic CSCR.

	Acute CSCR		Chronic CSCR		
—	Baseline (n = 38)	12 months (n = 12)	Baseline (n = 30)	12 months (n = 13)	
**SRF** ^a^ **(µm) mean** ± SD (range)	—	—	—	—	
All eyes	287 ± 221 (28–1,023)	31 ± 63 (0–215)	148 ± 134 (19–502)	40 ± 42 (0–143)	
Observation only	326 ± 236 (28–1,023)(n = 27)	36 ± 74 (0–215)(n = 9)	147 ± 149 (19–502)(n = 23)	18 ± 25 (0–52)(n = 8)	
Eyes with ≥50% SRF reduction (%)	—	7 (78%)	—	6 (75%)	
Eyes with zero SRF(%)	—	4 (44%)	—	5 (63%)	
Eplerenone therapy	192 ± 154 (53–570)	21 ± 41 (0–82)	150 ± 145 (45–267)	75 ± 42 (40–143)	
	(n = 11)	(n = 4)	(n = 7)	(n = 5)	
Eyes with ≥50% SRF reduction (%)	—	4 (80%)	—	2 (40%)	
Eyes with zero SRF(%)	—	3 (60%)	—	0 (0%)	
**Visual acuity decimal (snellen) mean** ± SD (range)	—	—	—	—	
All eyes	0.6 (20/32)±0.2	1.0 (20/20)±0.2	0.8 (20/25)±0.3	0.9 (20/22)±0.3	
	(0.2–1.0)	(0.8–1.25)	(0.2–1.25)	(0.3–1.25)	
Observation only	0.6 (20/32)±0.3	1.0 (20/20) ±0.2	0.8 (20/25) ±0.3	1.0 (20/20)±0.3	
	(0.2–1.0); (n = 27)	(0.8–1.3); (n = 8)	(0.2–1.3); (n = 23)	(0.3–1.3); (n = 8)	
Eplerenone therapy	0.7 (20/29) ±0.2	1.0 (20/20) ±0.2	0.8 (20/25)±0.2	0.9 (20/22)±0.3	
	(03–1.0); (n = 11)	(0.8–1.3); (n = 4)	(0.6–1.0); (n = 7)	(0.6–1.3); (n = 5)	

a SRF = subretinal fluid, n = number of eyes.

### Primary Outcome

Maximal height of SRF decreased from baseline to 12 months in acute [287 ± 221 (range 28–1,023) µm to 31 ± 63 (range 0–215) µm] and chronic [148 ± 134 (range 19–502) µm to 40 ± 42 (range 0–143) µm] CSCR.

### Secondary Outcomes

**FIGURE 2:**
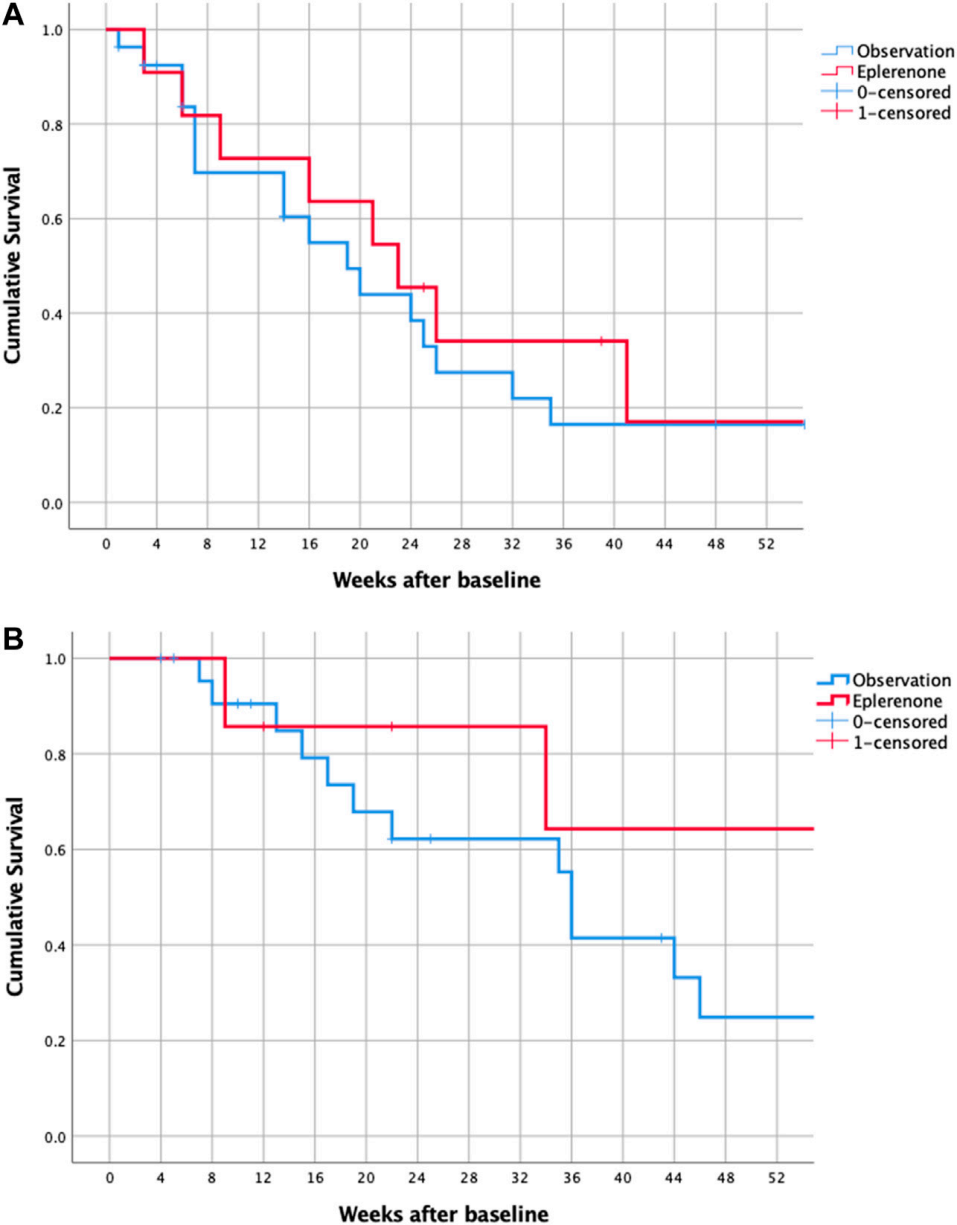
Kaplan-Meier survival of all eyes with acute **(A)** and chronic **(B)** CSCR reaching complete resolution of SRF (event = zero SRF). CSCR—central serous chorioretinopathy, SRF–subretinal fluid.

Percentages of eyes to reach complete resolution of SRF or achieve ≥50% reduction of SRF as well as VA are shown in table 1. Median time to complete resolution of SRF was 21 (12, 30) weeks for acute and 36 (24, 48) weeks for chronic CSCR. For both groups, acute and chronic CSCR, survival curves to reach complete resolution of SRF were similar for treated and observed eyes (Figure 2). COX regression analysis did not show a significant effect of eplerenone therapy on resolution of SRF: Acute CSCR [Exp(B) = 0.8 CI (0.3, 1.8)], *p* = 0.6; chronic CSCR [Exp(B) = 0.35 CI (0.1, 1.6)], *p* = 0.2.

Reasons for discontinuing eplerenone treatment included absence of anatomical or functional effect (1 eye), gastrointestinal side effects (2 eyes, 1 patient) and complete resolution of SRF (9 eyes). In 4 eyes, the reason for discontinuation could not be determined.

## Discussion

Aldosterone antagonists, specifically eplerenone, has been reported to be a pathophysiologically reasonable choice for treatment of CSCR(Zhao et al., 2012; Bousquet et al., 2013; Behar-Cohen and Zhao 2016). However, clinical results so far have been less convincing, and most recently, a well-designed double-blind, placebo-controlled trial (VICI) reported that eplerenone was not superior to placebo for chronic CSCR (Lotery et al., 2020b). Our real life data showing no effect of eplerenone on resolution of SRF nor improvement in VA in acute or chronic CSCR is consistent with the published data of the VICI trial.

In the majority of studies analyzing mineralocorticoid antagonists for CSCR, eplerenone was chosen due to its better safety profile compared to spironolactone. Its affinity in aldosterone-receptor blockage is 10–20-fold less than that of spironolactone *in vitro* (de Gasparo et al., 1987). However, it also has very low affinity to other steroid receptors, which significantly lowers progestational and anti-androgenic side effects and *in vivo* efficacy in blocking aldosterone-mediated changes in urinary Na:K ratio in rats is similar for both drugs (Delyani 2000). While comparative studies of eplerenone and spironolactone for its ophthalmological use are not conclusive, long lasting cardiological evidence of similar clinical efficacy and significant fewer side effects are definitely favoring eplerenone as drug of choice (Chin et al., 2015; Ghadiali et al., 2016; Kapoor and Wagner 2016; Pichi et al., 2016). Only 1 out of 18 patients in our study discontinued eplerenone due to side effects (i.e. gastrointestinal disturbance), confirming good tolerance of the medication.

The high percentage of spontaneous resolution of acute CSCR makes treatment indication in acute cases generally questionable (Liew et al., 2013). However, patients with large amounts of SRF are at higher risk for loss of photoreceptors and subsequent visual impairment, which could justify treatment for acute CSCR (Gerendas et al., 2018). In our study, all patients with acute CSCR were offered eplerenone as off-label therapy option. No difference in time to complete resolution of SRF could be shown between treated and observed eyes up to 12 months, even though baseline SRF of observed eyes was higher than baseline SRF of treated eyes (326 ± 236 vs. 192 ± 154 µm). Further, already at 3 months, zero SRF was achieved in similar proportion (36 and 30%) in treated and observed eyes respectively. This is contradictory to the only previous retrospective study comparing eplerenone treatment to observation in 22 eyes with acute CSCR (Zucchiatti et al., 2018). This study showed a higher resolution rate of SRF (80 vs. 25%) and a significant improvement in VA in the treatment group compared to observation only at 3 months (Zucchiatti et al., 2018). Further, another prospective study of 30 patients found accelerated resolution of SRF in patients treated with spironolactone compared to observation at 2 months (Sun et al., 2018). These diverging results could result from the small sample sizes, the short observation periods (2–3 months) as well as the different study drug with possible different efficacy in the study of Sun et al. Our data suggests that eplerenone does not accelerate SRF resolution in acute CSCR, however in the context of the limitations of our study (e.g. small sample size, variable follow-up time) and the sparse previous evidence, there are larger, prospective studies necessary to draw definite conclusions about eplerenone for acute CSCR.

In eyes with chronic CSCR, no difference could be detected between the treated and observed group in resolution of SRF or VA improvement. This coincides with the results of the VICI trial, where no difference in partial/complete resolution of SRF or VA was found at 12 months between eplerenone and placebo, but SRF decreased over time in both groups (Lotery et al., 2020b). Despite being the highest quality evidence on efficacy of eplerenone for CSCR, the VICI trial also has several limitations, i.e. possible introduction of bias by exclusion of eyes with secondary CNV without OCT angiography, non-balanced administration of additional PDT treatment between groups, and a possible ceiling effect on VA outcomes due to very good baseline VA (Lotery et al., 2020a; Sacconi et al., 2020; Stanescu-Segall et al., 2020; ). Other smaller prospective studies with variable patient selection, choice of aldosterone antagonist, controlling with placebo, and follow-up resulted in diverging results on functional and/or anatomical efficacy (Pichi et al., 2016; Rahimy et al., 2017; Schwartz et al., 2017). Due to the spectrum of CSCR and possible different response to treatment, drawing a definite conclusion needs to be done with caution (Daruich et al., 2016). However, our data does not suggest a relevant effect of eplerenone on resolution of SRF in chronic CSCR.

In chronic and acute CSCR, VA improved in both groups over time irrespective of treatment in our study. This again reflects the results of the VICI trial results. To note that VA might not be vicarious for treatment effect, since it mostly depends on the presence of subfoveal SRF and the state of the RPE and outer retina after its resolution (Liew et al., 2013).

Limitations of our study are its retrospective design and the possibility of selection bias (e.g. patients with eplerenone had a longer follow-up period than observation only eyes, eyes with longstanding SRF are more likely to get treatment) and the fact that there was no matching of patients. Further, comparison to other studies is difficult due to the varying definition of chronic disease (e.g. based on symptom duration ≥ 4 months in the VICI trial and others) and the non-standardized visits/treatment (Daruich et al., 2016; Lotery et al., 2020b). However, we believe to have chosen a pathophysiologically and clinically relevant distinction of acute vs. chronic CSCR with a combination of functional and structural parameters. There was a high number of loss to follow-up as is to be expected in a retrospective real-life study which needs to be accounted for in interpretation of the baseline and 12 months data. However, this does not affect the survival analysis.

In conclusion, the results of this study evaluating routine clinical practice data do not show any evidence of efficacy of eplerenone in resolution of SRF in CSCR (Lotery et al., 2020b).

## Data Availability

Data from this study will be shared upon request in an anonymized form.
