# Supplementary material for: Routine Clinical Practice Treatment Outcomes of Eplerenone in Acute and Chronic Central Serous Chorioretinopathy
Source: Front Pharmacol. 2021 May 10;12:675295. doi: 10.3389/fphar.2021.675295 (PMC8141746; doi:10.3389/fphar.2021.675295)
Supplement: Supplementary file 1 [file DataSheet1.docx]

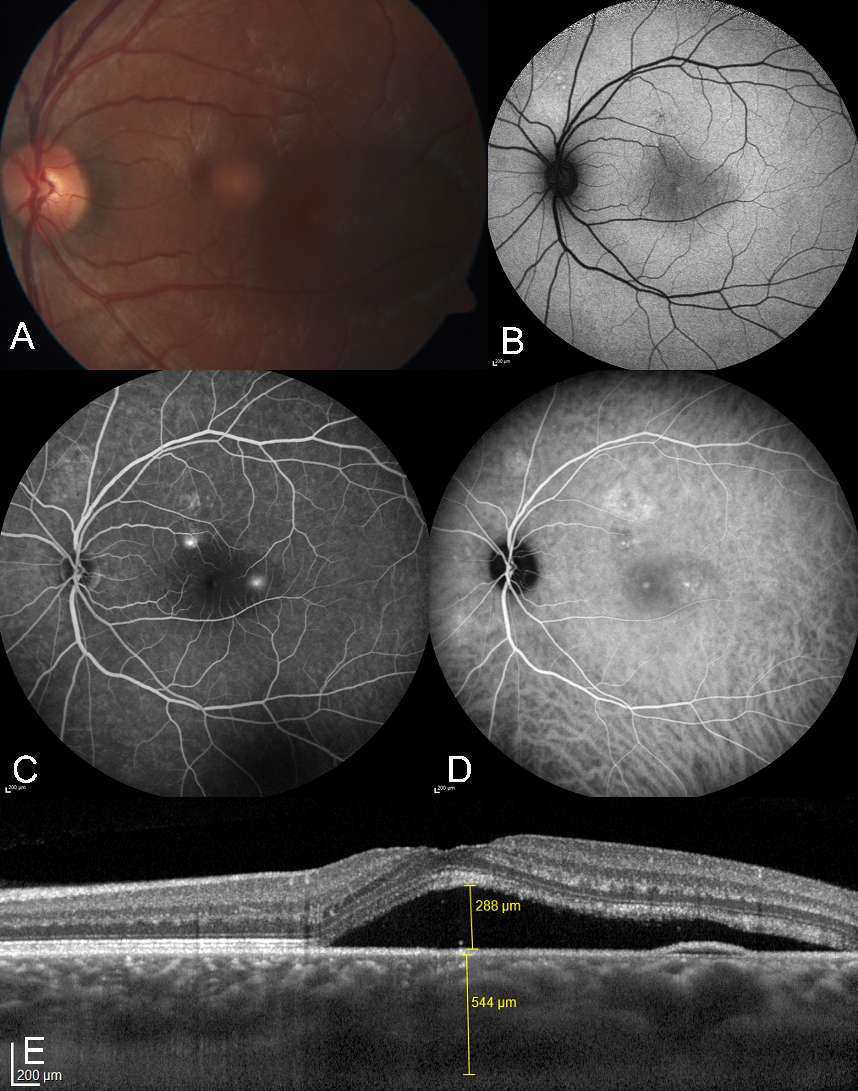


*Supplementary Figure 1: Example of acute CSCR, left eye, 52y♂*

A: Color picture with yellowish oval foveal retinal elevation. B: Slight focal hypoautofluorescence superior macula. C: Two “Ink blot” leakages on FA. D: corresponding focal hyperfluorescence on ICG. E: EDI-OCT demonstrating subretinal fluid, small serous RPE detachment, and increased choroidal thickness.

RPE – retinal pigment epithelium, FA - fluorescence-angiography, ICG - indocyanine-green angiography, EDI-OCT - enhanced depth imaging optical coherence tomography, SRF - subretinal fluid


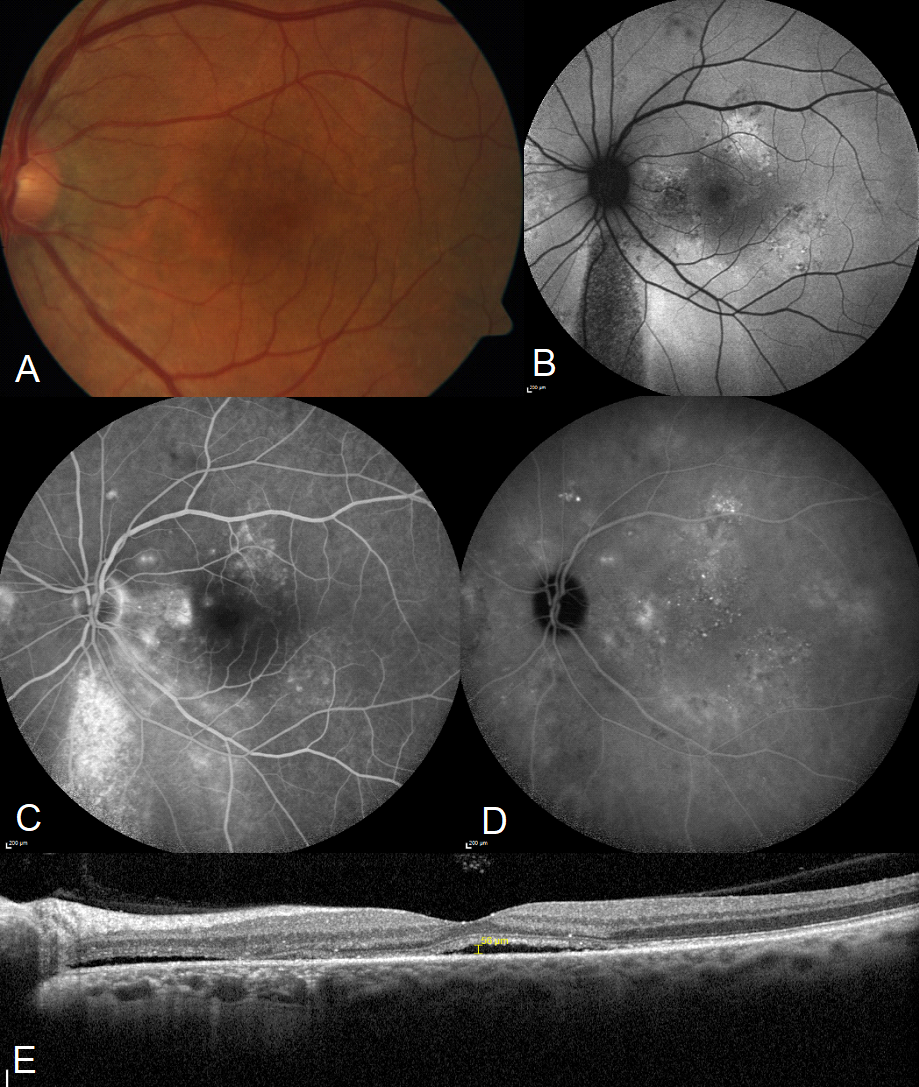


*Supplementary Figure 2: Example of chronic CSCR, left eye, 34y♂*

A: Color picture with RPE atrophy and hyperpigmentation. B: autofluorescence with hyper- and hypoautofluorescent descending tracts. C: FA showing spotty hyperfluorescence. D: ICG with choroidal hyperpermeabilities. E: EDI-OCT demonstrating subretinal fluid and increased choroidal thickness.

RPE – retinal pigment epithelium, FA - fluorescence-angiography, ICG - indocyanine-green angiography, EDI-OCT - enhanced depth imaging optical coherence tomography
